# Novel Double Endobutton Technique Combined with Three‐Dimensional Printing: A Biomechanical Study of Reconstruction in Acromioclavicular Joint Dislocation

**DOI:** 10.1111/os.12770

**Published:** 2020-08-19

**Authors:** Lei Zhang, Ai‐ni He, Yu‐feng Jin, Han‐wen Cheng, Lin Yu, Hua‐qiang Zhang, Jun‐jie Yao, Xin Zhou

**Affiliations:** ^1^ Department of Orthopaedics Affiliated Traditional Chinese Medicine Hospital of Southwest Medical University Luzhou China; ^2^ Center for Orthopaedic Diseases Research Affiliated Traditional Chinese Medicine Hospital of Southwest Medical University Luzhou China; ^3^ Academician Workstation Guangdong Province Medical 3D Printing Application Transformation Engineering Technology Research Center Luzhou China; ^4^ Clinical Base of Affiliated Traditional Chinese Medicine Hospital of Southwest Medical University Guangdong Province Medical 3D Printing Application Transformation Engineering Technology Research Center Luzhou China; ^5^ National Key Discipline of Human Anatomy School of Basic Medical Sciences, Southern Medical University Guangzhou China; ^6^ School of Clinical Medicine Southwest Medical University Luzhou China; ^7^ Department of Orthopaedics Luzhou Traditional Chinese Medicine Hospital Luzhou China

**Keywords:** Acromioclavicular joint dislocation, Guiding locator, Novel double Endubutton technique, TightRope technique

## Abstract

**Objective:**

To reconstruct the acromioclavicular (AC) joint using an adjusted closed‐loop double Endobutton technique *via* a guiding locator that was applied using three‐dimensional (3D) printing technology. At the same time, the reliability and safety of the novel double Endobutton (NDE) were tested by comparing the biomechanics of this technique with the TightRope (TR) approach.

**Methods:**

This retrospective study was conducted between January 2017 and January 2019. The Department of Anatomy at Southern Medical University obtained 18 fresh‐frozen specimens (8 left and 10 right; 12 men and 6 women). First, the guiding locators were applied using 3D printing technology. After preparation of materials, specimens were divided into an NDE group, a TR group, and a normal group. In the NDE and TR groups, the navigation module was used to locate and establish the bone tunnels; after that, the NDE or TR was implanted. However, the Endobuttons were fixed while pressing the distal clavicle downwards and the length of the loop could be adjusted by changing the upper Endobutton in the NDE group while the suture button construct was tensioned and knotted after pressing down the distal clavicle in the TR. Finally, load testing in anterior–posterior (AP), superior–inferior (SI), and medial–lateral (ML) directions as well as load‐to‐failure testing in the SI direction were undertaken to verify whether the NDE or TR had better biomechanics.

**Results:**

In the load testing, the displacements of the NDE and TR groups in the AP, SI, and ML direction were significantly shorter than those of the normal group (*P* < 0.05). In the load‐to‐failure testing, the ultimate load of the NDE and TR groups had significantly higher increases than the normal group (722.16 ± 92.04 *vs* 564.63 ± 63.05, *P* < 0.05; 680.20 ± 110.29 *vs* 564.63 ± 63.05, *P* < 0.05). However, there was no statistically significant difference between the two techniques for these two tests (*P* > 0.05). In the NDE group, four of six failures were a result of tunnel fractures of the coracoid, while two of six were due to suture breakage. In the TR, three failures were due to coracoid tunnel fractures, one was a result of a clavicle tunnel fracture, and the rest were due to suture breakage. In the normal group, half of the failures were a result of avulsion fractures of the conical ligament at the point of the coracoid process, and the other three were due to rupture of the conical ligament, fracture of the distal clavicle, and fracture of the scapular body.

**Conclusion:**

As for the TR technique, the stability and strength of the AC joint were better in patients who underwent reconstruction using the NDE technique than in the intact state.

## Graphical abstract

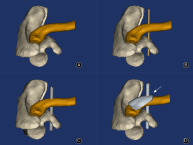

This study created a novel double Endobutton technique combined with 3D printing and verified that its biomechanics were not statistically different from those of TR but were better than for normal ligaments.

## Introduction

Acromioclavicular dislocation (ACD) is one of the most common orthopaedic problems and accounts for 9% of all shoulder injuries^1^, ^2^. High‐grade injuries (Rockwood IV and above) require surgery. A variety of surgical techniques have been reported to reestablish the anatomical acromioclavicular (AC) joint alignment. However, there is still no gold standard surgical approach^1^, ^2^, ^3^, ^4^, ^5^, ^6^.

Early surgical procedures applying Kirchner wires or Steinman pins are no longer used due to the case reports of pin migration and the limitation of relative motion between the scapula and clavicle ^2^, ^3^, ^4^, ^5^, ^6^. The hook plate through the acromion requires a secondary operation for implant removal and increases the risk of AC ligament injury^7^, ^8^. Conjoined tendon transfer reconstructing the static and dynamic stability of the AC joint is currently used for old ACD. The surgery decreases the incidence of redislocation, while increasing the risk of coracoid and brachial plexus injury ^9^, ^10^.

On the basis of the Weaver–Dunn procedure, which uses the native AC ligament in AC joint reconstruction, there are many surgeries that can be performed to reconstruct the coracoclavicular (CC) ligament through tendon grafts or plate fixation^10^, ^11^. The TightRope (TR) technique, involving two connected buttons and FiberWire sutures, is a common fixation surgery^12^, ^13^. This technique prevents the CC ligament from separating and maintains the stability of the AC joint. However, the models are prone to failure because of suture breakage^12^, ^14^.

The Endobutton technique, which aims to reconstruct the CC ligament and restore the physiological function of the AC joint, has become a common surgery in the clinical treatment of ACD. Struhl^15^ first described the double Endobutton technique in 2007, to minimize joint subluxation and fractures. Struhl demonstrated that the stability of the AC joint can be maintained by placing a suture button construct between the coracoid process and the distal clavicle, which was greater than the nature ligament in strength and stiffness. As for the TR, there is no need to remove the fixation devices for reoperation. For long‐term success without construct slippage, Struhl *et al*.^16^ created a closed‐loop double Endobutton to reconstruct both acute and chronic dislocations. They suggested that this technique without rigid fixation had better stability in both the axial and superior directions than a cortical button, which may benefit from the healing of soft tissue.

To provide an excellent bone strut for the closed‐loop double Endobutton and to restore the anatomical direction of the AC ligament, some research demonstrates that the key to this technique is to construct the clavicle–coracoid tunnel precisely and quickly. The most important factor in determining the optimal location and diameter of the bone tunnel accurately is the attachment of the native CC ligament to the clavicle and the coracoid process^17^, ^18^, ^19^, ^20^. Sella *et al*.^20^ reported that the TR technology also required a higher tunnel position to maintain the consistency of the directions of the clavicle and the coracoid process, so that the incidence of suture breakage could be reduced. Nonetheless, the clavicular–coracoid tunnels constructed currently using the navigation system are not completely consistent with the AC joint, which may result in a long‐term operation, intraoperative hemorrhage, a poor tunnel, or even fracture of the coracoid^21^, ^22^.

In recent years, 3D printing technology, as an emerging biofabrication technology, has been widely used to mimic natural 3D models in *in vitro* tissue research, especially as it is becoming more financially feasible and accessible to use in orthopaedic surgery. 3D printing technology provides anatomical details of various bones and tissues. The 3D printer can not only build a real size model, which can help orthopaedic surgeons diagnose diseases, but also make the implantation of plates quicker and more accurate, reducing surgical complications, and make it easier to check whether the plate fits well^23^.

Apart from the problems of transosseous tunnels, another serious limitation of the NDE is that the length of the loop is determined before the procedure, which is approximately equal to the CC interval in an uninjured shoulder. This may result in difficulty controlling tension and intraoperative mismatch, for instance, so that the operative time may be prolonged, and the efficiency of the operation could be reduced.

As a consequence, this study created a novel double Endobutton (NDE), which could adjust the length of the closed loop according to actual situations intraoperatively, to reconstructed the AC joint. In the meantime, a guiding locator was applied using the 3D printing technique to locate and establish the bone tunnels. Furthermore, we compared the displacements in the anterior–posterior (AP), superior–inferior (SI), and medial–lateral (ML) directions, at the loads of 5 N, 20 N, 40 N, and 60 N, as well as the ultimate loads of the NDE in the SI direction with the TR technique and normal ligaments.

The purpose of this study was: (i) to improve the efficiency of the closed‐loop double Endobutton technique; (ii) to make the AC joint reconstruction more precise, so that the incidence of complications could be reduced; and (iii) to provide evidence and support for clinical extensive application of the novel technique.

## Materials and Methods

### *Inclusion and Exclusion Criteria*

From January 2017 to January 2019, 18 fresh‐frozen shoulder specimens (8 left and 10 right; 12 men and 6 women) were obtained by the Department of Anatomy of the Southern Medical University. Bone density had been determined by X‐ray to be a normal specimen (OSTEOCORE‐3, Golden, China).

The inclusion criteria were as follows: (i) full‐grown and normal Chinese AC joint; (ii) the CC ligament of the AC joint had been removed; (iii) magnetic resonance imaging of the AC joint without intact CC ligament; (iv) manipulative reduction had failed to treat AC dislocation; and (v)had received anatomical reconstruction of the AC joint. The exclusion criteria were as follows: (i) incomplete specimens; (ii) musculoskeletalor soft tissue injury in the AC or shoulder joint (e.g. osteoarthritis, shoulder instability, or fracture); and (iii) congenital malformation of the AC joint. All of the specimens were wrapped with gauze soaked in physiological saline, and stored at 24°C (a cryogenic freezer was provided by Southern Medical University). Before biomechanical testing, the specimens were thawed at room temperature for 12 h.

### *Three‐Dimensional* *Printing* *of Guiding* *Locator*

Images of the AC joint were acquired prior to soft tissue dissection to preserve the anatomic alignment of the bones and joint space width. The images were obtained using a 16‐slice spiral CT scanner (Siemns, Germany) with 0.8‐mm pitch, 120 kV, and 120 mAs, and imported into Mimics 17.0 (Materialize NV, Leuvan, Belgium). Using the Mimics software, the 3D model (0.75‐mm slice thickness and 0.75‐mm reconstruction interval) of the AC joint was established and saved in Mimics Document (.mcs) format. The 2D model of the NDE was built with SolidWorks 2017 (SolidWorks, USA). Using the stretch, cut, and other functions, the NDE was transformed from a 2D to a 3D model.

**Fig 1:**
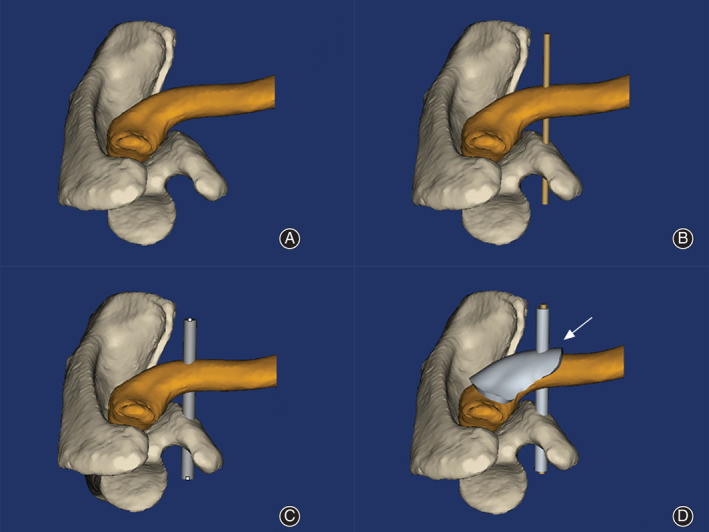
The design of the guiding locator. (A) The anterior view of the acromioclavicular joint model. (B) A light pipe passing through the clavicle and the coracoid process. (C) The acromioclavicular joint model with a connecting rod. (D) The acromioclavicular joint model with guiding locator.

In the Mimics software 3D interface, the location, direction, and length of the bone tunnel were determined using the NDE first, which were selected by drawing lines along the bone surface of the AC joint. Next, a tunnel with the same diameter as the Kirschner wire was established along the preestablished direction. Then the position and direction of the navigation was adjusted to align with the virtual tunnel by selecting a convergence point (Fig. 1). Based on the module, a virtual personalized ACD bone tunnel navigation can be obtained through the “Boolean operation” to firmly fix the AC joint. Finally, using biodegradable plastic polylactic acid, the locator data was generated by 3D printer (MakerBot Replicator 2, USA) to create a personalized entity.

### *Materials Preparation*

**Fig 2:**
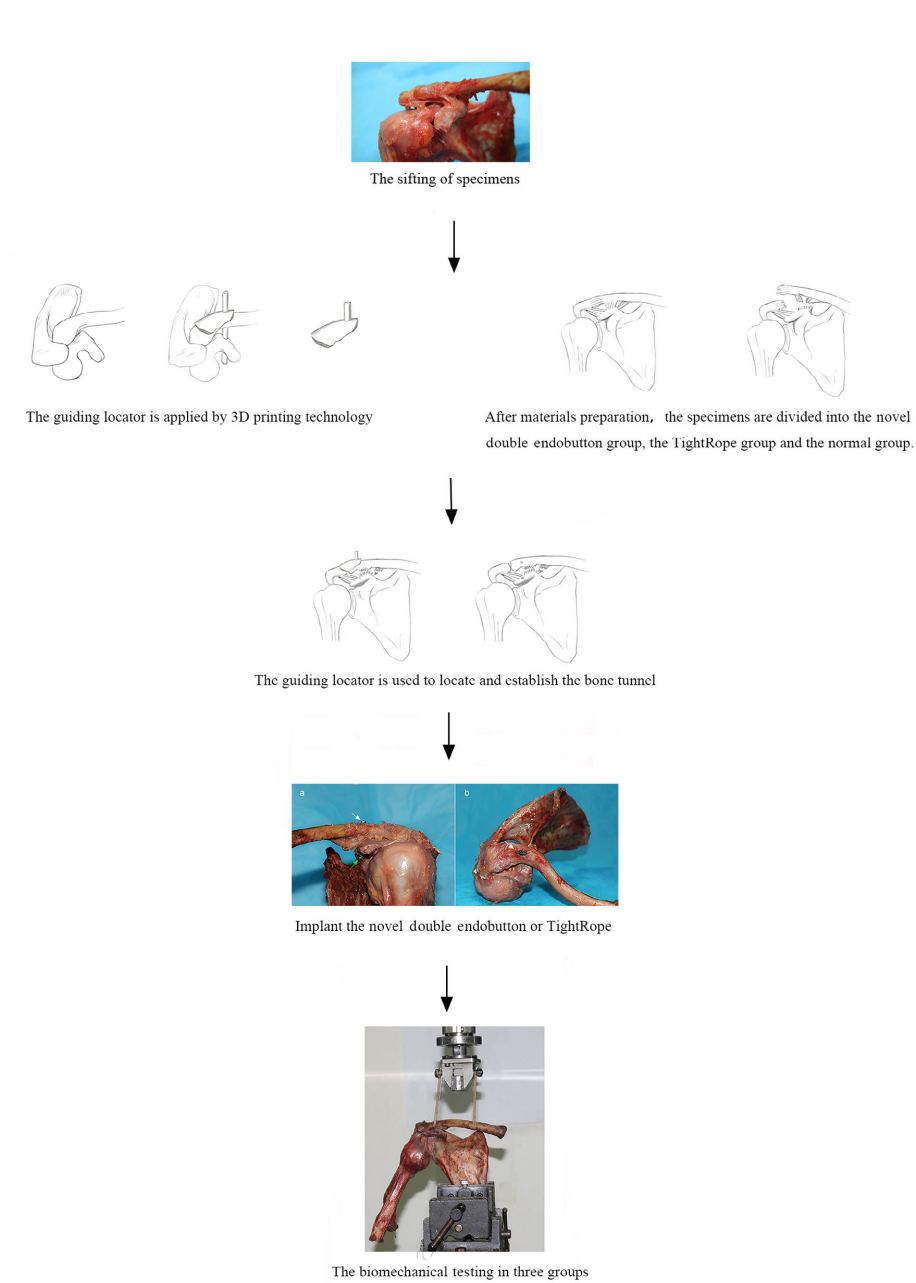
Three‐dimensional model and picture of the novel double Endobutton.

The specimens were divided into three groups: an NDE group, a TR group, and a normal group. All specimens underwent primary AC joint reconstruction according to the different groups, illustrated in Fig. 2. The soft tissues of each specimen were removed, except for the AC joint capsule, the AC ligament and the CC ligament complex. After removing the scapula, we exposed the CC construction completely, including the distal clavicle by approximately 4 cm, the coracoid, the CC ligament complex (the conoid and trapezoid ligament), and the AC joint.

After anatomical observation and measurement, the AC ligament and the CC ligament were cut off with a scalpel in the NDE and TR groups (Rockwood III or above), and the ACD model was constructed. The specimens were placed in a special clamp to ensure the stability of the models during the experiment.

### *Surgical Procedure for the Novel Double Endobutton Technique*

First, two 1.5–2.0‐cm marks were made 2 cm medial to the distal end of the clavicle and 4 cm medial to the distal end of the clavicle, which was consistent with anatomical attachment landmarks of the trapezoid (women, 1.6 to 2.7 cm and men, 1.7 to 2.8 cm) and conoid ligaments (women, 2.9 to 4.4 cm and men, 3.4 to 5.0 cm)^4^, ^24^.

Second, after the guiding locator was inserted from the anterior–medial approach and located at the center of the base of the coracoid and the center of the upper surface of the clavicle, a 1.5‐mm Kirschner wire was implanted between these two points. The 4.5‐mm bone tunnels were drilled using the same diameter bit along the Kirschner wire.

**Fig 3:**
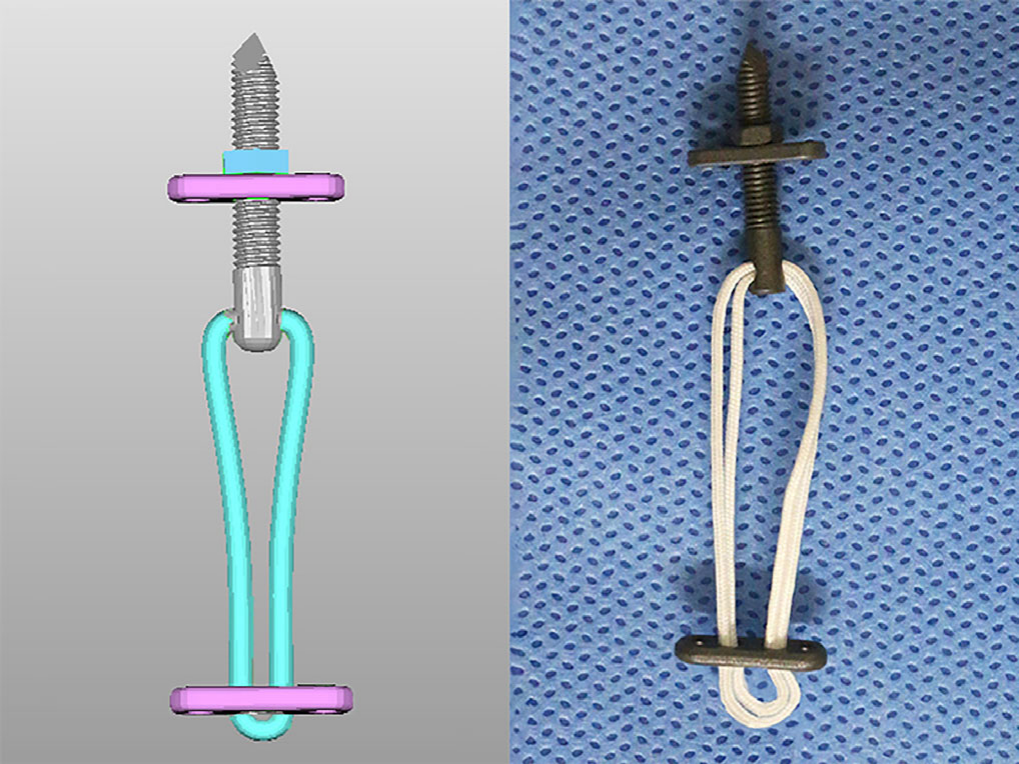
The restoration and fixation of the novel double Endobutton. The white narrows show the upper Endobutton plate, and the green narrow shows the lower Endobutton plate. (A) The left acromioclavicular joint in anterior view. (B) The superior aspect of the left acromioclavicular joint.

**Fig 4:**
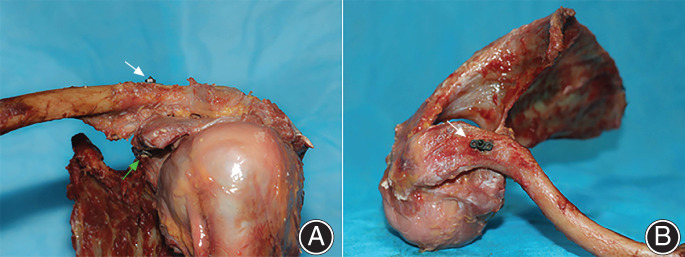
Specimen with the novel double Endobutton mounted on the test machine for load‐to‐failure testing in superior–inferior direction.

Third, the adjustable closed‐loop double Endobutton technique (Fig. 3, Delta Medical, China) was used to reconstruct the anatomic course of the CC ligament: One of the Endobuttons (12‐mm length, 4‐mm width, and 1‐mm thickness, titanium alloys) was taken from the clavicle tunnel to the base of the coracoid tunnel using a lead suture. After twisting the Endobutton, the lower button was fixed on the base of the coracoid and the upper button was fixed on the top of the clavicle while pressing the distal clavicle downwards. The length of the loop (Ethicon 5, a high polymer polyethylene) could be adjusted according to the situation by changing the upper Endobutton, and the knot of the closed loop could be formed for locking and resetting. After that, the lead suture on the Endobutton was drawn out (Fig. 4).

### *Surgical Procedure of the* *TightRope* *Technique*

The method was similar to the NDE technique. It was only after pressing down the distal clavicle to restore the AC joint that the suture button construct was tensioned and knotted.

### *Load Testing*

**Fig 5:**
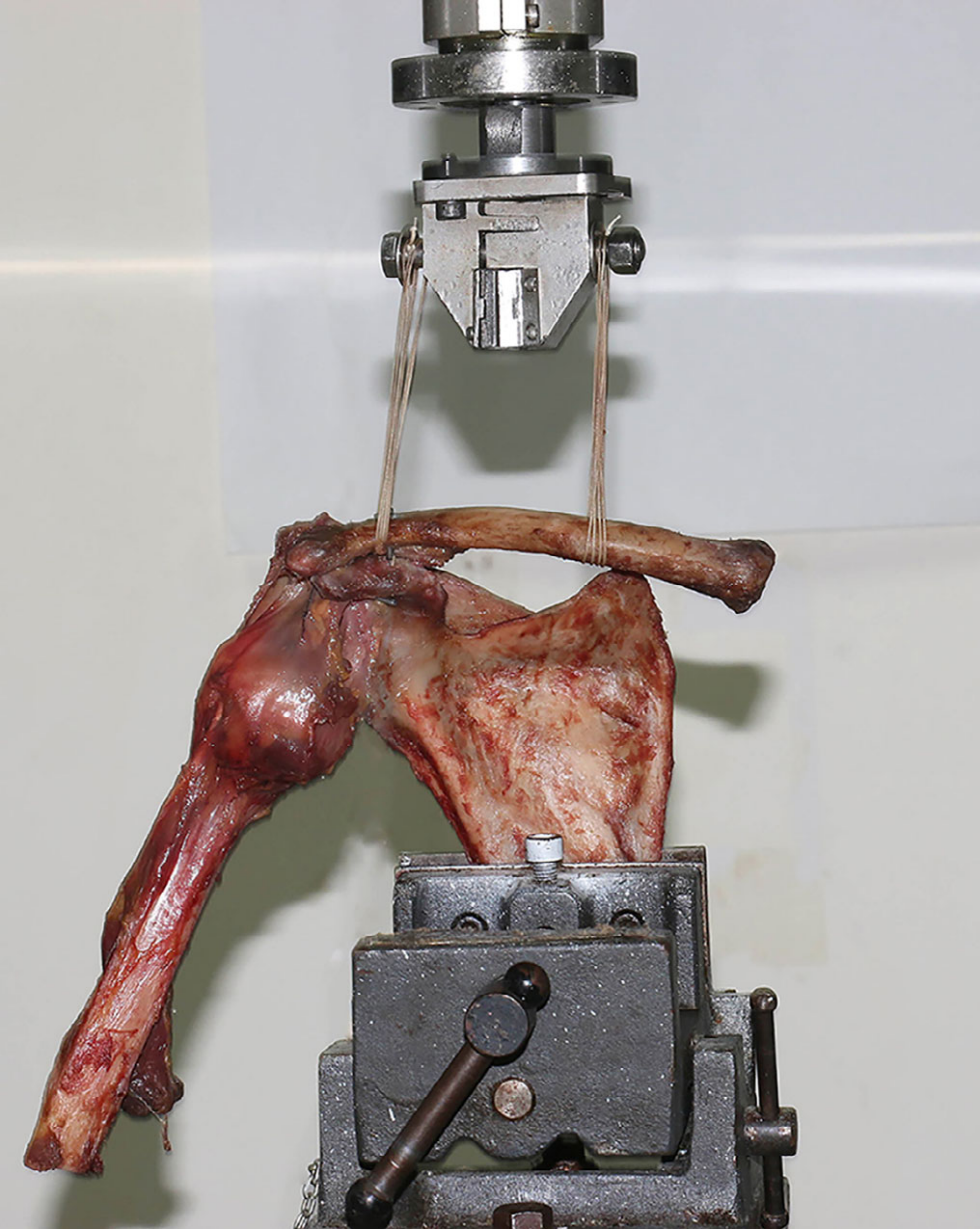
Basic principle flowchart, including three‐dimensional printing of the guiding locator, preparation of materials, the surgical procedure, and the biomechanical test.

First, three groups of specimens were taken from the bone–ligament–bone structure. Both ends of the bone structure were fixed with a special fixture; one end was connected and fixed to the dynamic unit of the biomechanical testing machine (Bose Electro Force 3520‐AT, USA) and the other end was connected and fixed to the fixing unit of the testing machine. Then, both ends of the AC joint were carefully connected with an electronic grating ruler and fixed in a special jig. The proximal clavicle was vertically oriented in its neutral anatomic position relative to the scapula by adjusting the jig; this orientation permitted sequential loading in the plane along the axis of the AC joint and perpendicular to the AC joint line (Fig. 5)^25^.

In this research, 70 N, which is below the threshold of osseous bending and simulates the stresses of mild physical rehabilitation^25^, ^26^, was applied as the upper limit of the vertical load to prevent irreversible damage to the specimens caused by repeated loading. Before the initial loading, a static superior preload (5 N) was carried out to eliminate the time effect, stress relaxation, and creep of bone or ligaments; the distance at 5 N was set as the initial reference (starting point). The preloading was slowly removed before the formal test. At the same time, the specimens were kept moist with saline solution.

The vertical load in the testing was 5–70 N. The biomechanical testing machine was applied to the load at a constant speed of 5 N/s. The specific loads were: 5 N, 20 N, 40 N, and 60 N. The experiment was repeated 10 times, and the loading interval of each test was kept above 2 min to relieve the stress fatigue of specimens. The stress‐deformation curve in the AP, SI, and ML directions was recorded by testing the deformations and displacements of the ligaments under the corresponding strength, respectively.

### *Load‐to‐Failure Testing*

After being reoriented, the specimens with intact CC ligament constructs were fixed to a biomechanical machine. The experiments were performed at the constant speed of 1 mm/min in the SI direction until rupture of the CC ligament, internal fixation failure, or avulsion fracture. The load–displacement curves and ultimate loads at the time of construct failure were recorded by the computer linked to the biomechanical machine.

### *Studying Indexes*

#### *The Displacement*

The degree of displacement of the AC joint was determined by measuring the CC interval distance and calculating the variables of the CC interval. The CC interval distance was measured from the clavicle anterior–inferior border and the coracoid process superior border. Mean values were recorded in millimeters. The CC interval distance was determined based on standard anteroposterior views of the AC arch in both preoperative images and those taken at loads of 5 N, 20 N, 40 N, and 60 N for each shoulder.

#### *The Ultimate Load*

The ultimate load, defined as the highest load in the SI direction when the model failed, was measured using a biomechanical machine. As reported in the literature, the ultimate failure load of the intact CC ligament in isolation was 500–725 N^27^.

### *Statistical Analysis*

Data were collated by the computer linked to the biomechanical machine, and analyzed using SPSS 20.0 statistical software. In this research, the stability of the AC joint was expressed by the variables of the CC interval (displacement), and the ultimate loads represented the strength of the NDE or the TR. Importing the collected data into the computer in Microsoft Office Excel 2016 format, the data of different states and positions were calculated and analyzed statistically. All measurements were expressed as mean ± standard deviation. The homogeneity of variance was performed by using the Shapiro–Wilk test. One‐way ANOVA was used to compare the three groups, with a *P*‐value <0.05 considered statistically significant.

## Results

### *Load Testing*

The load testing findings are summarized in Table 1. In any direction, the displacements under all loading conditions were significantly shorter for the normal group compared with NDE and TR groups (*P* < 0.05). There was no statistically significant difference between the two techniques (*P* > 0.05).

**Table 1:** The load and load‐to failure results (mean±SD)

Groups	5 N	20 N	40 N	60 N	Ultimate load (N)
Anterior–posterior					
Normal	0.39 ± 0.27	1.29 ± 0.72	4.12 ± 2.32	7.71 ± 3.98	—
Endobutton	0.14 ± 0.06*	0.59 ± 0.31*	1.36 ± 0.79*	2.06 ± 1.25*	—
TightRope	0.09 ± 0.05*	0.60 ± 0.45*	1.89 ± 1.75*	2.96 ± 2.40*	—
*F‐*value	5.77	3.70	4.26	7.15	
*P*‐value	0.014	0.049	0.034	0.007	
Superior–inferior					
Normal	0.30 ± 0.31	0.97 ± 0.62	1.67 ± 0.77	2.37 ± 0.88	564.63 ± 63.05
Endobutton	0.07 ± 0.03*	0.41 ± 0.20*	0.85 ± 0.37*	1.31 ± 0.53*	722.16 ± 92.04†
TightRope	0.06 ± 0.03*	0.30 ± 0.16*	0.70 ± 0.37*	1.13 ± 0.61*	680.20 ± 110.29†
*F‐*value	3.43	5.16	5.64	5.67	4.87
*P*‐value	0.059	0.020	0.015	0.015	0.023
Medial–lateral					
Normal	0.24 ± 0.05	0.96 ± 0.31	2.06 ± 0.80	3.29 ± 1.32	—
Endobutton	0.12 ± 0.06*	0.55 ± 0.22*	1.12 ± 0.48*	1.87 ± 1.04*	—
TightRope	0.15 ± 0.08*	0.55 ± 0.16*	1.10 ± 0.29*	1.64 ± 0.50*	—
*F‐*value	5.17	6.19	5.71	4.69	
*P*‐value	0.020	0.011	0.014	0.026	

^*^ *P* < 0.05 *vs* Normal group.

† *P* < 0.05 *vs* Normal group.

In the normal group, the acromioclavicular (AC) and coracoclavicular (CC) ligaments remained. The Endobutton was the novel double Endobutton technique.

These dashes indicated that no texting was conducted.

### *Load‐to‐failure Testing*

The ultimate load of the NDE or TR technique had a significantly higher increase from the normal state (722.16 ± 92.04 *vs* 564.63 ± 63.05, *P* < 0.05; 680.20 ± 110.29 *vs* 564.63 ± 63.05, *P* < 0.05). However, there was no statistical difference between these two techniques either (*P* > 0.05).

The failures of the normal group were various: three were avulsion fractures of the conical ligament at the point of the coracoid process; the other three were, respectively, rupture of the conical ligament, fracture of the distal clavicle, and fracture of the scapular body. In the NDE group, the modes of failure included coracoid fracture through the drill hole in four specimens and suture breakage in two specimens. In the TR group, the modes of failure were coracoid fracture through the drill hole in three specimens, clavicle tunnel fracture in one specimen, and suture breakage in two specimens.

## Discussion

### *Load Testing*

The stability for the novel double Endobutton technique and the TightRope technique were better than that of the normal state, with smaller displacements in AP, SI, and ML directions. However, there was no statistically significant difference between these two techniques.

The NDE and TR reconstruction using a closed loop has been shown to have satisfactory biomechanical and clinical outcomes^26^, ^28^. The closed loop was used to maintain the integrity of the models. It not only maintained the rotation of the AC joint by non‐rigid fixation but also eliminated the possibility of knot slippage, especially the adjustable closed loop in NDE, and avoided the intraoperative mismatch problem in TR. At the same time, it reduced the redundant steps, such as resetting and knotting, so that the operation time was shortened. The length of the loop can be adjusted according to the actual situation intraoperatively, which can improve the surgical efficiency and ensure the accuracy of the operation. Peeters *et al*.^26^ showed that the closed loop with sutures above and below the joint roughly doubled the contact surface, which was slightly more resistant to displacements and reached higher maximal forces by reducing initial cutting of the suture in the superior and inferior plane.

For both techniques, the anatomical structure of the clavicular–coracoid–acromion was reconstructed using suture button construct. As in Celik *et al*.^29^, the acromion and distal clavicle were perfectly aligned in neutral alignment and could prevent superior translation. The construct ensured the stability of the AC joint. However, we designed the construct without any muscle tissues and AC ligaments bridging the span^4^, ^21^, ^30^, ^31^, ^32^, which may cause large displacements.

### *Load‐to‐Failure Testing*

Load‐to‐failure testing of both techniques showed excellent ultimate loads in all specimens, which demonstrated that the strength of both techniques was better than for the natural ligaments. In this study, with the stress displacement gradually increased, the CC ligaments of the normal group first showed a fibrous tear and, finally, ruptured, while both of the techniques failed mainly because of tunnel fractures.

Numerous studies have demonstrated that the strength of the suture button construct is better than that of the natural ligaments^13^. Chernchujit *et al*.^27^ reported the ultimate tensile strength of a two‐anchor system with FiberWire No. 5 that resulted in a value of 767 ± 109 N, exceeding the tensile strength of the native CC ligaments (578 ± 111 N), which was enough to maintain vertical stability and provide early mechanical stability for ligament healing and cicatrization. Harris *et al*.^33^ developed forces for CC sling, asuture anchor, and acoracoclavicular screw; only the bicortical coracoclavicular screw showed a tensile strength of 927 N. In Takase *et al*.^34^, the strength of the construct was more than 40% of the natural ligaments. Others reported that the loop had more than double the strength (1063 N) but similar stiffness (142 N) to the normal ligaments^16^. Some studies report that the endobutton can withstand anywhere from 1086 to 1365 N^35^, while the ultimate load of TR can exceed 1400 N^36^. However, none of the groups in this study reached the ultimate load reported in the literature. This could be due to the bone‐ligament‐bone structure includes the properties of the clavicle and coracoid, and the clavicle stiffness would be reduced by 40% after dislocation^36^, ^37^, ^38^, ^39^.

Previous studies have stated that the risk of fracture is highly correlated with the use of bone tunnels, especially the position and the size of the tunnels. Spiegl *et al*.^40^ demonstrated that the size of the clavicular can impair the bone and increase the risk of fractures. The number and size of coracoid drill holes would affect stability and increase the incidence of fractures^28^, ^32^, ^41^. Struhl showed that the shear forces in either the axial or AP direction contributed to the risk of not only suture abrasion but also tunnel widening. A larger amount of widening was correlated with increased AP and rotational laxity^16^, ^25^. Thus, the anatomical position of the sutures and buttons has a crucial role against rotational and shear forces^42^, ^43^.

In this study, the AC joint model was reconstructed by dual‐source CT. At the same time, the optimal location of the clavicle–coracoid tunnel and the clavicular navigation module was designed using digital optimization technology. The module was printed simultaneously with 3D printing technology to further verify the accuracy and reliability. Using the guiding locator could not only avoid a situation such as iatrogenic fractures and the injury of nerves and vessels but also mean that individuals are treated more accurately and faster. As stated by Dyrna *et al*, it could optimize tunnel placement and further reduce the risk of these technical problems^29^, ^30^.

### *Limitations*

Several limitations of the present study should be considered. First, although the NDE designed in this study had many advantages, it was larger in size and needs to be further improved. Second, the soft tissues of specimens in this study were removed. This did not take into account many of the deformable forces on the shoulder and arm during reconstruction. Third, the movement of AC joint has various directions and amounts of forces that may change the results in all models. However, only gravity force without any additional forces was used for the analyses and the models examined in this research. Therefore,the double endobutton technique and the procedure used to verify biomechanics could ideally be optimized in future. At the same time, multiplanar ultimate loads could be evaluated.

### *Conclusion*

The stability and strength of the novel double Endobutton technique with a guiding locator for AC joint reconstruction were better than in the intact state. In contrast to the TightRope technique, there was no significant difference. In this study, the majority of failures for both techniques were fractures.
